# Molecular Profiling of Malignant Pleural Effusions with Next Generation Sequencing (NGS): Evidence that Supports Its Role in Cancer Management

**DOI:** 10.3390/jpm10040206

**Published:** 2020-11-01

**Authors:** Georgia Ι. Grigoriadou, Stepan M. Esagian, Han Suk Ryu, Ilias P. Nikas

**Affiliations:** 1 1st Department of Medical Oncology, Theageneio Anticancer Hospital, 54007 Thessaloniki, Greece; georgiag678@gmail.com; 2 Department of Medicine, School of Health Sciences, Democritus University of Thrace, 68100 Alexandroupolis, Greece; stepesag@gmail.com; 3 Department of Pathology, Seoul National University Hospital, Seoul 03080, Korea; karlnash@naver.com; 4 School of Medicine, European University of Cyprus, 2404 Nicosia, Cyprus

**Keywords:** liquid biopsy, supernatant, pleural fluid, non-small cell lung carcinoma, NSCLC, precision oncology, cytology, EGFR, therapy resistance, biomarkers

## Abstract

Malignant pleural effusions (MPEs) often develop in advanced cancer patients and confer significant morbidity and mortality. In this review, we evaluated whether molecular profiling of MPEs with next generation sequencing (NGS) could have a role in cancer management, focusing on lung cancer. We reviewed and compared the diagnostic performance of pleural fluid liquid biopsy with other types of samples. When applied in MPEs, NGS may have comparable performance with corresponding tissue biopsies, yield higher DNA amount, and detect more genetic aberrations than blood-derived liquid biopsies. NGS in MPEs may also be preferable to plasma liquid biopsy in advanced cancer patients with a MPE and a paucicellular or difficult to obtain tissue/fine-needle aspiration biopsy. Of interest, post-centrifuge supernatant NGS may exhibit superior results compared to cell pellet, cell block or other materials. NGS in MPEs can also guide clinicians in tailoring established therapies and identifying therapy resistance. Evidence is still premature regarding the role of NGS in MPEs from patients with cancers other than lung. We concluded that MPE processing could provide useful prognostic and theranostic information, besides its diagnostic role.

## 1. Introduction

Pleural effusion (PE), the pathologic accumulation of excess fluid inside the pleural cavity, is a complication that often appears in a wide spectrum of clinical settings. Prevalent underlying causes include non-neoplastic conditions (e.g., congestive heart failure and infections) and cancer, either metastatic or primary [1,2]. Lung and breast cancers make up the most common malignancies that spread into the pleural surfaces forming malignant pleural effusions (MPEs) [3,4]; the latter appear in around 16% of patients with M1b Stage IV non-small cell lung cancer (NSCLC) [5]. In contrast, mesothelioma, a primary pleural malignancy linked to asbestos exposure, is a relatively rare cause of MPE compared to metastases [3,4,6]. MPEs confer significant morbidity, mortality, and poor survival rates, while their management is primarily palliative, intending to ease symptoms and improve quality of life [3,7].

Laboratory examination of MPEs provides robust diagnostic, prognostic, and theranostic information [8]. As soon as a PE specimen arrives to the pathology laboratory for processing, it undergoes centrifugation, which divides the fluid into a cell pellet (cellular-rich material) at the bottom of the tube and a supernatant lying above it. The cell pellet is subsequently used to form diverse preparations including direct smears, cytospins, liquid-based cytology (LBC) slides and/or formalin-fixed paraffin-embedded (FFPE) cell blocks; these are stained and evaluated morphologically to formulate a diagnosis [2,9]. Ancillary techniques such as Immunohistochemistry (IHC) and Fluorescence in situ Hybridization (FISH) are routinely applied on any of the aforementioned preparations to solve selective diagnostic issues—e.g., metastatic adenocarcinoma vs. mesothelioma differential diagnosis; confirmation of the primary malignancy site or provide clinically relevant prognostic and predictive information; examples of the latter include HER2 testing in breast cancer and PD-L1 testing to initiate immunotherapy [2,9,10]. Notably, similar to tissue biopsies, isolation and further processing of DNA, mRNA, miRNAs, and proteins are highly effective on MPEs [2,8,10]. The latter comprise an optimal material for analysis techniques such as PCR and sequencing, while molecular testing can successfully be performed on all previously mentioned cell pellet-based preparations besides effusion supernatants [2,8,9,10,11].

In the era of precision medicine, testing MPEs against selected genetic aberrations can assess prognosis and guide patient selection for established targeted therapies [8,10]. Concerning advanced NSCLC, the most recent ASCO guidelines recommend each tumor to be tested for EGFR, ALK, ROS1, and BRAF aberrations in any adequate and well-preserved biopsy or cytology specimen, as molecular therapies exist for all aforementioned targets. Genes including KRAS, MET, RET, and ERBB2 could also be included when multiplex molecular testing is chosen. In addition, cellular material should be kept for PD-L1 IHC before immunotherapy [12,13]. Although single gene molecular analysis makes up the most commonly applied approach, it could often pose problems, especially when applied to cytology samples [9]. Pathologists must triage the already scant cytologic material into multiple diagnostic, prognostic, and theranostic tests, while, as they need to render cytomorphologic diagnosis and perform essential ancillary testing (IHC, FISH), they should treat any leftover with caution. In this setting, serial single-gene testing could exhaust the remaining material and prove insufficient for precision medicine, when multiple biomarkers need to be checked such as with NSCLC [14,15]. Next generation sequencing (NGS) can simultaneously detect and quantify, in a massive parallel and high throughput manner, multiple genomic alterations such as point mutations, insertions, deletions, gene fusions, and amplifications in multiple specimens. Of interest, NGS only needs a single DNA or RNA input from each sample, sparing precious material that has high analytical sensitivity, while it provides comprehensive molecular coverage and is cheaper per base compared to sequential single biomarker testing [16,17,18,19].

Draining a PE is a minimally invasive procedure, while a MPE might sometimes be the only specimen available for analysis, especially when tissue biopsy is paucicellular or impossible to retrieve in the setting of advanced unresectable cancer [10,20,21,22]. Of interest, disseminated tumor cells (DTCs), cell-free DNA (cfDNA), circulating tumor DNA (ctDNA), exosomes, and other secretory vesicles can be detected in PEs similar to plasma liquid biopsy [2,11]. Besides tissue biopsies and plasma liquid biopsy, molecular analysis on MPEs also has the potential to guide personalized cancer management. As the literature is far more extensive for liquid biopsies from blood plasma/serum of cancer patients compared to other biological fluids [23,24], this manuscript aims to review the published evidence related to the application of NGS in MPEs.

## 2. Pleural Fluid Liquid Biopsy Compared to Other Types of Samples

Among all sources of genetic material, tissue biopsy is considered the current standard for the molecular characterization of tumors and pre-analytical factors (such as the DNA/RNA concentration and quality) can heavily influence the results of the molecular analysis [25]. Of interest, Zhang et al. found that the DNA extracted from pleural fluid FFPE cell blocks had similar quality to its tissue counterpart, while freshly centrifuged pleural fluid preparations achieved even higher quality standards [26]. Yamamoto et al. also described comparable RNA concentrations between pleural fluid and tissue samples [27].

The aforementioned pre-analytical properties may account for the highly accurate results following MPE NGS analysis. Xiang et al. and Liu et al. reported concordance rates between pleural fluid and tissue NGS samples of 83.3% (50/60 mutations) and 86.7% (26/30 mutations), respectively [28,29]. Zhang et al. successfully detected EGFR mutations in 15/15 previously confirmed cases [30]. Song et al. identified EGFR mutations and ALK aberrations in the pleural fluid of 68/123 and 11/123 of the tested patients, respectively, while the EGFR wild-type was associated with a PD-L1 IHC score ≥ 50%. Of interest, pleural fluid and tissue samples had a concordance rate of 86.2% (25/29) for PD-L1 IHC expression with a 50% threshold; this directly translates to identifying patients that could benefit from pembrolizumab therapy [31,32]. In many studies, a large proportion of discordant mutations between pleural fluid and tissue were actually novel mutations not detected in the original tumor biopsy [26,28,29,33,34]. Like other types of liquid biopsy, these mutations may accurately reflect intratumoral (spatial and temporal genetic) heterogeneity, rather than being false positive results, thus providing a more complete picture of the tumor’s mutational landscape [35]. In some cases, these mutations had direct implications for patient management, as they were potentially targetable or resistance-conferring to targeted molecular agents [26,33].

Liquid biopsy is predominantly performed via plasma analysis, while cerebrospinal fluid (CSF) and pleural fluid are gaining increasing popularity as alternative sources of genetic material. Plasma, specifically, offers the advantage of easy retrieval through a routine blood draw. On the other hand, pleural fluid collection requires thoracentesis, which is a procedure with more complications [36]. Tong et al. and Villatoro et al. reported that pleural fluid samples had higher cfDNA concentrations and mutation allele frequencies (MAFs) compared to plasma samples [24,37], while Liao et al. showed that pleural fluid NGS detected more unique mutations compared to other sample types, including plasma [34]. Zhang et al. found a concordance of 86.7% between the pleural fluid and plasma NGS among 15 patients harboring known EGFR mutations; two mutations were missed by plasma analysis while all mutations were successfully detected by pleural fluid analysis [30].

Overall, these data suggest that pleural fluid may be a more reliable source of genetic material than plasma, given its superior pre-analytic indices and mutation-detecting ability. Despite its slightly more invasive nature, pleural fluid liquid biopsy might be able to offer a more reliable alternative to the classic tissue biopsy, whilst avoiding many of the shortcomings of plasma liquid biopsy [35]. Pleural fluid also offers the additional advantage of cytologic examination. Fine needle aspiration (FNA) is another source of genetic material suitable for NGS that also provides this option. However, Zhang et al. showed that its DNA concentration and quality was lower compared to that of pleural fluid [26]. The main findings of the studies described in this section are summarized in Table 1.

## 3. Correlation of Pleural Fluid NGS with Cytomorphologic Findings and Tumor Cellularity

In a study of patients with various malignancies, Yang et al. noticed a correlation between the results of cytology and NGS. Specifically, at least one mutation was detected in every sample with confirmed (9/9) or suspicious (2/2) cytology for malignancy, while no mutations were found in benign effusions (0/4) [39]. The same authors found a significant correlation between tumor cellularity and the variant allele frequency [40]. Leichsenring et al. also noted many false negative results when tumor cellularity dropped below 10% [38]. These findings suggest that cytologic evaluation is crucial to guide molecular sequencing. Samples completely lacking malignant cells are less likely to detect mutations, while samples without sufficient tumor cellularity may be prone to false negative results due to inadequate allele frequencies. In these cases, physicians may opt for a sample of higher tumor cellularity instead, thus saving valuable resources from redundant molecular analyses while simultaneously obtaining a more accurate picture of the tumor’s mutational profile. Interestingly, the MPE volume did not correlate with tumor cellularity and therefore low MPE volume should not deter NGS analysis, provided that tumor cellularity is sufficient [41].

Nonetheless, there are reports of adequate mutation detection even with unfavorable pre-analytic characteristics. Buttitta et al. showed that NGS was able to detect 70% (7/10) of tissue-confirmed EGFR mutations in matched pleural fluid samples of low tumor cellularity (<10%). In comparison, Sanger sequencing was only able to detect 20% (2/10) of mutations, showcasing the advantages of deep sequencing over conventional sequencing methods [42]. Liu et al. found that mutations were successfully detected in samples with even lower tumor cellularity (<5%) in 85.7% (6/7) of cases, thus making an argument for using pleural fluid specimens with a non-ideal pre-analytic profile [29]. However, false positive results may arise under those circumstances, as germline or clonal hematopoietic mutations may be mistaken for tumor mutations [39]. The same phenomenon has been described in plasma liquid biopsy [35]. As a solution, Yang et al. suggests that paired white blood cells should be simultaneously sequenced to exclude mutations of non-tumor origin [39]. The abovementioned information is summarized in Table 2.

## 4. The Value of Supernatant-Derived cfDNA

Cell blocks are the most common source of genetic material when performing pleural fluid molecular analysis. Τheir preparation requires centrifugation of the sample, followed by retrieval and special processing of the sediment, while the supernatant is usually discarded [2,9,28]. However, recent evidence suggests that the cfDNA found in the supernatant can also be successfully used for molecular analysis with comparable, if not superior, results to cell blocks. Yang et al. showed that 100% (8/8) of the mutations found in FFPE cells blocks were also detectable in the matched supernatant of three NSCLC patients [39]. Xiang et al. found that pleural fluid supernatant NGS had higher concordance with tissue samples and yielded more known mutations compared to FFPE cell blocks [28]. Li et al. reported comparable mutational profiles and MAFs between supernatant and FFPE cell blocks for all genes included in the current diagnostic recommendations for NSCLC [43]. In addition, the MAFs from pleural fluid aspirate were even higher when compared to cell blocks in isolated cases [44]. Aside from high accuracy, supernatant analysis had a much shorter turnaround time compared to cell block preparation, which can last up to a week [28].

An alternative approach to cell block preparation is the direct extraction of genomic material from sedimentary tumor cells following centrifugation. In a study by Zhang et al., all known EGFR mutations (15/15) were successfully detected in both supernatant and sedimentary tumor cells, but the former achieved much higher MAFs [30]. Similarly, Tong et al. found that both sample types had comparable sensitivity (93% [27/29] with supernatant vs. 90% [26/29] with sediment-derived DNA) in detecting tissue-confirmed driver mutations. However, the supernatant displayed superiority in multiple other analytical indices, many of which had direct implications for treatment. These include achieving higher MAFs, tumor mutational burden, chromosomal instability, and sensitivity in locally metastatic patients, as well as higher detection of resistance-conferring (e.g., EGFR T790M) and unique mutations in advanced cancer patients, and more driver mutations in tissue-lacking patients. In addition, the supernatant retained its sensitivity in cases of cytologically-negative (86%) or hemorrhagic pleural fluid (72%), whereas the sensitivity of sedimentary tumor cells was significantly compromised (9% vs. 30% decrease in sensitivity, respectively) [37]. Overall, these findings suggest that supernatant analysis provides a more accurate depiction of the tumor mutational landscape and may be a more reliable source of genetic material when the sample tumor cellularity is either low or mixed by abundant non-neoplastic cellular elements.

Tumor exosomes are nucleic acid-containing microvesicles released into the tumor environment, thus providing a novel non-cellular source of genomic material for molecular analysis. Song et al. compared tumor exosome to supernatant cfDNA NGS analysis and found a concordance of 77.9% (243/312). Importantly, the concordance for ALK and EGFR mutations across 18 patients was 100%, showing that tumor exosomes derived from pleural fluid can be used to guide treatment with targeted agents. Another important observation was that the concordance of the two sample types increased to 94.1% (128/136) when copy number variations (CNVs) were excluded from the analysis [45]. Liao et al. has attributed the inherent weakness of cfDNA to detect CNVs to its fragmentation [34]. In addition, Tong et al. reported that supernatant-derived cfDNA revealed more CNVs (31%, 20/64) compared to sedimental tumor cell-derived DNA (14%, 9/64) [37]. Therefore, this shows that the sensitivity of supernatant in detecting CNVs via cfDNA is limited, thus alternative sources of genetic material could potentially be sought in this situation. The abovementioned information is summarized in Table 3.

## 5. Evaluation of Therapeutic Resistance, Response, and Management

cfDNA from (MPEs) can provide diagnostic information and reveal mutations in molecular pathways associated with therapy resistance. This information could direct the therapeutic management of NSCLC patients [38,46]. Common mutations associated with resistance to targeted therapy are EFGR T790M and ALK p.G1202R. Yang et al. and Villatoro et al. were able to isolate these mutations from MPEs of patients with disease progression after first-line treatment with tyrosine kinase inhibitors (TKIs). These results successfully guided therapeutic decisions and eventually led to a clinical benefit [24,39]. Zhang et al. discovered the EGFR T790M mutation in two NSCLC patients via MPE NGS, with one of them representing a novel mutation undetected in the matched tissue sample [26]. Goldberg et al. described a case of a patient progressing on third line TKIs, where pleural fluid NGS was utilized to detect a novel C797S mutation responsible for treatment failure [47]. Similarly, Li et al. reported a case for which MPE NGS was utilized to determine the cause of crizotinib non-response [48]. Other less common molecular biomarkers, including RET, HER2, and MET, can reveal additional therapeutic targets [12]. Wang et al. revealed MET amplifications in the pleural fluid of two patients who were subsequently treated with crizotinib based on this result [33]. Tong et al. found that there was no difference in the progression-free survival of 10 patients, when treatment decision with TKI was based on pleural fluid supernatant rather than tissue analysis [37].

Culturing cells from MPEs could be an alternative way for guiding therapeutic management. An example is choosing candidates for polyADP-ribose polymerase inhibitor (PARPi) therapy, which requires the presence of homologous recombination DNA repair (HRR)-defective cancer cells [49]. Patterson et al. successfully determined the HRR mutational status of patients using MPE-derived cell lines. Their sample included four NSCLC patients, three of whom had defective HRR, thus showing that selecting candidates for PARPi therapy using this alternative approach was feasible [50]. Using a similar approach, Roscilli et al. tested the in vitro response of MPE-derived cultured cell lines to different chemotherapy regimens and were able to successfully predict the patients’ clinical response. This approach also allowed them to select regimens that had a synergistic effect, thus optimizing treatment selection. Interestingly, the same findings could not be reproduced in regard to TKIs, indicating that additional genetic or epigenetic factors may play a role in determining the response to these targeted molecular agents [51]. The main findings of these studies are summarized in Table 4.

## 6. The Role of NGS in Malignant Pleural Effusions from Different Types of Cancers (Other than Lung)

There is a growing interest on the role of MPE analysis by NGS outside of lung cancer as well [38]. In two patients with breast cancer and colorectal cancer, Yang et al. found that their MPE supernatant NGS was concordant with their matched lymph node FNA cell block and surgical tissue NGS, respectively [39]. Shah et al. showed that MPE NGS in three metastatic ovarian cancer patients successfully revealed tissue-confirmed TP53 mutations. In two cases, TP53 MAFs were higher in pleural fluid compared to its matched tissues (FFPE and frozen), indicating higher tumor DNA fraction [53]. Similar to lung cancer, cases of novel mutations in MPE analysis have also been described with other malignancies. Using MPE-derived cultured cell lines from a patient with disseminated medulloblastoma, Xu et al. were able to detect a novel 17q deletion that was not previously detected in the tissue sample. After culturing cell lines from the tissue, the mutation was eventually confirmed, proving that it was also present in the tissue and that the FFPE tissue analysis results were false-negative [54]. This finding highlights the ability of pleural fluid to capture theumor heterogeneity more accurately and provides new insights for the applications of cell cultures in tumor molecular analysis. Finally, Zhou et al. showed that pleural fluid could potentially serve as an alternative to other liquid biopsy types, such as plasma and ascitic fluid, with comparable results in a case report of a gastric cancer patient [55]. A summary of these studies could be found in Table 5.

## 7. Discussion

**Figure 1:**
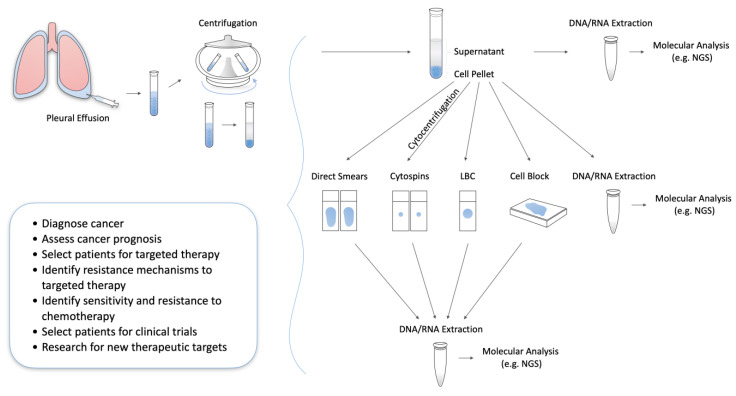
Pleural fluid processing to facilitate diagnosis, prognosis, and guide therapy of metastatic cancers. NGS = Next generation sequencing, LBC = Liquid-based cytology.

MPEs from patients with advanced cancers are often processed in pathology laboratories for rendering diagnoses, assessing prognostic factors, and selecting patients for established precision therapies [8,10], Figure 1.

As MPEs could be the first and only specimen received, especially when tissue biopsies are hypocellular or impossible to obtain, their efficient triage and processing is imperative to get maximum clinically relevant information [10,20,21,22]. Besides the necessary morphologic evaluation and routine ancillary preparations, MPEs can provide robust theranostic information similar to tissue biopsies. Both cell pellets—including all preparations derived from it like direct smears, LBC slides, and FFPE cell blocks—and post-centrifuge supernatants can subsequently be processed for molecular testing [2,14,15]. This is critical for cases like advanced NSCLC, where testing for multiple biomarkers is recommended to tailor targeted oncologic therapies, according to the latest ASCO guidelines. NGS, a molecular technique that can detect multiple genomic aberrations in a single run, can successfully be applied in effusions [16,17,18,19]. Most published studies deal with advanced NSCLC; however, research has also shown promise in the application of NGS in MPEs caused by breast, colorectal, ovarian, gastric, and small cell lung cancers, as well as melanoma (Table 5) [24,38,39,53,55].

Recent evidence suggests that NGS in MPEs is highly concordant with NGS in correspondent tissue biopsies, whereas the former might even detect additional driver and resistance aberrations, highlighting intratumoral heterogeneity (Table 1) [26,28,33,37]. In contrast to tissue biopsies, isolating genetic material from effusions is not hampered from artifacts linked with formalin fixation (except for cell blocks), such as the crosslinking between nucleic acids and proteins [15,56].

A growing number of studies point to testing plasma liquid biopsies to guide clinical management in advanced cancer patients, especially when tissue biopsy is insufficient or impossible to obtain [24,26,37,39]. Similar to plasma yet with a superior performance, MPE liquid biopsy allows multiplex molecular testing to identify driver and resistance mutations, disease monitoring through serial collection, and optimal view of tumor heterogeneity in advanced cancer patients [8,19,57].

For any NGS analysis to be successful, standardization of pre-analytical factors is imperative for optimal results [2,16,19,25]. In cytologic specimens like MPEs, pre-analytical factors include sample adequacy, fixatives, preservatives, diverse cytopreparation methods, staining, tumor fraction, nucleic acid extraction protocols, and DNA/RNA input [19,25]. NGS performance seems to deteriorate when cellularity is low, yet it is more effective than Sanger sequencing in hypocellular contexts [38,42]. Of interest, effusion-derived supernatant—a material normally discarded during fluid preparation —has provided robust molecular analysis results [9,15], exhibiting high concordance to tissue and superior performance to plasma liquid biopsy. Apart from MPEs, literature has also shown optimal results in supernatants derived from fine needle aspirations (FNAs) of diverse cancers [15,58,59]. Normally, we need to assess tumor cellularity before every molecular test, yet this is impossible to do when utilizing supernatants. However, the latter contain plenty of ctDNA derived from the high turnover of cancer cells, the active or passive release through necrosis and apoptosis, and potentially the disruption of cancer cells during centrifugation [9,15,60].

Apart from standardizing pre-analytic factors, implementing a complex, high-throughput procedure such as NGS in a clinical laboratory requires additional steps. After selecting the most suitable platform, gene panel, and enrichment method, each NGS assay needs to be validated, as it is imperative to establish its diagnostic performance (e.g., analytical sensitivity and specificity) [18]. Within this framework, laboratory personnel should optimize a workflow that includes library preparation, sequencing, and big data analysis [16,19]. Data mining begins with base-calling and continues with sequence alignment, variant detection (e.g., single nucleotide variants, indels, copy number variants, and gene rearrangements), and annotation, before reporting to the patient. To carry out this pipeline, it is necessary to closely collaborate with bioinformaticians, who apply sophisticated algorithms and filter background “noise” [16,18,19]. Of interest, a bigger database is created with each NGS run, allowing comparisons besides the discovery of novel genetic aberrations. However, reporting incidental findings of unknown clinical significance to the patient carries significant ethical and legal implications [18]. Except for the challenge to implement a complex procedure that generates such massive amount of data into laboratories that routinely perform low-throughput testing, management and storage of big data derived from NGS creates significant bioethics dilemmas related to the protection of patient privacy [61].

## 8. Conclusions

In conclusion, molecular analysis of MPEs with NGS is a powerful, high throughput modality able to identify targetable biomarkers, stratify for established targeted therapies and clinical trials, and pinpoint mechanisms of therapy resistance. Pleural fluid liquid biopsy is minimally invasive and often permits repeated follow-up, while it captures tumor heterogeneity more efficiently than tissue biopsy. Evidence suggests that NGS in MPEs has comparable performance with tissue and is more effective than plasma liquid biopsy, especially when post-centrifuge supernatants are used. In addition, NGS exhibits superior results to Sanger sequencing in hypocellular fluid specimens. Despite the aforementioned promising results, evidence is still premature and potentially misleading, as it mostly comes from small and, to a great extent, retrospective studies of low power or case reports. In this context, large studies in the form of randomized controlled trials would be of significant value. Furthermore, validation is still needed at both pre-analytical and analytical levels. Pathologists should triage MPEs efficiently, as multiple diagnostic, prognostic, and theranostic tests need to be performed from each sample. For instance, the cell pellet could be used for subsequent morphologic evaluation and routine ancillary techniques including HER2 or PD-L1 IHC, while the supernatant should not be discarded albeit saved for high-throughput molecular analysis, especially when the cellular material runs out. Lastly, when tissue biopsy or FNA are paucicellular or impossible to retrieve and a MPE is formed, molecular analysis of the latter can be favored over plasma liquid biopsy in advanced cancer patients.

## Figures and Tables

**Table 1:** NGS analysis of NSCLC pleural fluid liquid biopsy samples compared with tissue biopsies and FNA, plasma, CSF, and BAL samples.

First Author/Reference	Pleural Fluid Material	Summary of Findings
Zhang et al. [26]	Cell blocks	➢ Pleural fluid cell blocks had similar DNA quality, while fresh pleural fluid had higher DNA quality compared to tissue biopsies.
		➢ Pleural fluid samples had lower cellularity, but higher DNA concentration and DNA quality index compared to matched FNA samples from the primary site.
		➢ A TKI resistance-conferring mutation (EGFR T790M) was detected in a pleural fluid sample but not in its matched tissue sample.
Yamamoto et al. [27]	N/A	➢ Pleural fluid had similar RNA concentration with tissue biopsy and BAL.
Xiang et al. [28]	Supernatants, Cell blocks	➢ Pleural fluid supernatant NGS showed 83.3% concordance (50/60) with tissue molecular analysis.
Liu et al. [29]	Cell blocks	➢ Pleural fluid supernatant NGS showed 86.7% (26/30) concordance with tissue molecular analysis.
Zhang et al. [30]	Supernatants, sDNA	➢ Pleural fluid supernatant NGS showed 100% sensitivity (15/15 mutations) in detecting previously confirmed EGFR mutations.
		➢ Pleural fluid supernatant NGS showed 86.7% concordance (13/15) with plasma molecular analysis.
Tong et al. [37]	Supernatants, sDNA	➢ Pleural fluid supernatant molecular analysis showed higher cfDNA concentration, MAFs, chromosomal instability, and TMB and detected more CNVs, unique mutations, driver mutations, and drug resistance-conferring mutations compared to matched plasma samples.
		➢ Pleural fluid supernatant molecular analysis showed similar MAFs, chromosomal instability, and TMB compared to matched tissue samples.
Villatoro et al. [24]	Supernatants	➢ Pleural fluid supernatant molecular analysis showed higher cfDNA concentration and MAFs compared to matched plasma and CSF samples.
Liao et al. [34]	Supernatants, Cell pellets	➢ Pleural fluid NGS from both supernatants and cell pellets detected less SNVs per patient compared to matched plasma and tissue samples.
		➢ Pleural fluid NGS samples showed the highest mutation frequency for 29.1% (14/48) of the tested genes.
		➢ Pleural fluid NGS detected unique mutations not found in plasma or tissue molecular analysis.
Leichsenring et al. [38]	Cell blocks	➢ Cell block NGS showed 100% concordance (2/2) with tissue in two patients.
Wang et al. [33]	N/A	➢ MET amplifications were revealed in two patients progressing on TKIs post-treatment (undetected in pre-treatment tissue).
		➢ While detected in the pre-treatment tissue sample, pleural fluid NGS missed EGFR del19 mutation in one patient.
Yang et al. [39]	Supernatants	➢ More mutations were revealed by post-treatment pleural fluid compared to matched pre-treatment lymph node FNA molecular analysis.

NGS: next-generation sequencing, NSCLC: non-small cell lung cancer, FNA: fine needle aspiration, CSF: cerebrospinal fluid, BAL: bronchoalveolar lavage, sDNA: sediment DNA, MAF: mutation allele frequency, TMB: tumor mutational burden, CNV: copy number variations, cfDNA: cell-free DNA, SNV: single nucleotide variation, N/A: not available, TKI: tyrosine kinase inhibitor.

**Table 2:** Correlation of pleural fluid NGS with cytomorphologic findings and tumor cellularity.

First Author/Reference	Pleural Fluid Material	Summary of Findings
Yang et al. [39]	Supernatants	➢ All malignant (9/9, five of which were from NSCLC) and suspicious (2/2, one of which was from NSCLC) pleural fluid samples revealed mutations.
		➢ No mutations were found in benign samples.
Yang et al. [40]	Cell blocks	➢ A significant correlation between tumor cellularity and VAF was revealed.
Leichsenring et al. [38]	Cell blocks	➢ Pleural fluid NGS showed false negative results in samples with low tumor cellularity (<10.0%).
Buttitta et al. [42]	Cell blocks	➢ In low cellularity pleural fluid samples (0.3–7.0%), 70% (7/10) of mutations were detected by NGS compared to 20% (2/10) by Sanger sequencing.
		➢ In pleural fluid samples without malignant cells, only 20% (1/5) of mutations were detected by NGS and no mutations were detected by Sanger sequencing.
Liu et al. [29]	Cell blocks	➢ NGS on pleural fluid samples with low tumor cells (<5%) revealed concordant mutations with matched tissue samples in 85.7% (6/7) of cases.
Carter et al. [41]	Cell blocks	➢ No relationship was found between malignant pleural effusion volume and pleural fluid overall or tumor cellularity.

NSCLC: non-small cell lung cancer, NGS: next-generation sequencing, VAF: variant allele frequency.

**Table 3:** The value of supernatant-derived cfDNA.

First Author/Reference	Pleural Fluid Material	Summary of Findings
Xiang et al. [28]	Supernatants, Cell blocks	➢ Pleural fluid supernatant NGS revealed 89.1% (41/46) of somatic mutations vs. 54.3% (25/46) somatic mutations in cell blocks.
		➢ Low concordance was found between supernatant and cell block, tissue and cell block, but high concordance was found between supernatant and tissue NGS.
		➢ Supernatant analysis had a much shorter turnaround time compared to cell block preparation, which can last up to a week.
Yang et al. [39]	Supernatants, Cell blocks	➢ Pleural fluid supernatant NGS showed 100% concordance (8/8 mutations) with cell block molecular analysis derived from the same pleural fluid specimens of three patients.
Li et al. [43]	Supernatants, Cell blocks	➢ Comparable mutational profile and MAFs were found between supernatant and cell block NGS for all genes currently recommended for mutational testing.
Wei et al. [44]	Aspirate, Cell blocks	➢ Higher MAFs were detected in the pleural fluid compared to cell block NGS in one patient.
Zhang et al. [30]	Supernatants, sDNA	➢ Supernatant NGS showed 100% concordance (15/15) of EGFR mutations with sedimentary tumor cells in 15 patients.
		➢ Higher MAFs of supernatant were found compared to sedimentary tumor cell NGS.
Tong et al. [37]	Supernatants, sDNA	➢ Pleural fluid supernatant NGS showed similar sensitivity (93%, 27/29 mutations) with sDNA (90%, 26/29) for driver mutations; two tissue mutations were missed in all samples.
		➢ Supernatant NGS had higher MAFs, chromosomal instability, and tumor mutational burden compared to sDNA NGS.
		➢ Supernatant NGS had higher sensitivity for tissue-determined mutations of M1a patients compared to sDNA NGS.
		➢ Supernatant had more total driver mutations (43) vs. sDNA (38) in 31 patients.
		➢ More EGFR T790M mutations were detected in supernatant (5/5) vs. sDNA (3/5).
		➢ More unique mutations were detected in supernatant vs. sDNA for M1b/c patients.
		➢ A non-significant sensitivity reduction (82% to 71%) was noted with hemorrhagic pleural effusion in supernatant compared to a significant sensitivity reduction in sDNA (64% to 34%).
		➢ Supernatant had a sensitivity of 86% for cytologically-negative samples, compared to sDNA that only had 9%. Supernatant remained superior even after excluding these cases (*p* = 0.027).
		➢ 31% (20/64) of CNVs were detected in supernatant vs. 14% (9/64) in sDNA NGS.
Song et al. [45]	Supernatants, Tumor exosomes	➢ 77.9% concordance (243/312 mutations) were found between PE cfDNA and PE exoDNA NGS.
		➢ The concordance increased to 94.1% (128/136 mutations) after excluding CNVs.
		➢ The concordance for EGFR and ALK mutations in 18 patients was 100%.
Liao et al. [34]	Supernatants, Cell pellets	➢ CNVs of 17 targetable genes were detected with cell pellet in 66.0% (31/47) of patients but not with supernatant NGS (unreadable due to fragmentation).

NGS: next-generation sequencing; NSCLC: non-small cell lung cancer; CNV: copy number variation; sDNA: sediment tumor DNA; MAF: mutation allele frequency; PE: pleural effusion, exoDNA: exosomal DNA.

**Table 4:** NGS in MPE for the evaluation of therapeutic resistance, response, and management.

First Author/Reference	Pleural Fluid Material	Summary of Findings
Leichserning et al. [38]	Cell blocks	➢ Clinically actionable mutations were detected that guided targeted therapy.
DiBardino et al. [46]	Cell blocks, slides	➢ Mutations were detected in 4 out of the 5 patients whose pleural fluid samples were tested. These mutations changed management in two of these patients.
Yang et al. [39]	Supernatants	➢ Resistance mutations were revealed in 2 patients; EGFR T790M and ALK p.G1202R were detected in two patients previously treated with erlotinib and crizotinib, respectively.
Villatoro et al. [24]	Supernatants	➢ T790M was detected in pleural effusion supernatant NGS of two patients progressing on 1st/2nd generation TKIs and used to guide clinical decision. Clinical benefit was observed in both.
Zhang et al. [26]	Cell blocks	➢ EGFR T790M mutation was detected in pleural fluid but not in tissue NGS of one patient (cancer heterogeneity).
Goldberg et al. [47]	N/A	➢ C797N was found in pleural fluid NGS of one patient who had previously progressed on 2nd-line TKI with C797S mutation initially.
Li et al. [48]	N/A	➢ NGS in a patient with no response to crizotinib revealed EML4-ALK fusion (25.3%), CDK2NA del, and TP53 mutation.
Wang et al. [52]	N/A	➢ Pleural fluid NGS revealed MET amplification which guided targeted therapy (crizotinib).
Tong et al. [37]	Supernatants, sDNA	➢ Pleural fluid supernatant NGS results were used to tailor TKI-based treatment in 10 patients who subsequently exhibited comparable PFS.
Patterson et al. [50]	Cell cultures	➢ Homologous recombination repair was revealed in 12/13 cell lines. This information can be used to stratify patients for either PARPi or platinum therapy.
Roscilli et al. [51]	Cell cultures from cell pellets	➢ Patients with the same mutational profile (EGFR del19, without T790M) exhibited different sensitivities to the same TKIs in two cultures (NGS not designed to detect epigenetic aberrations).
		➢ In vitro chemosensitivity testing for classic chemotherapeutic agents matched with clinical response to treatment.
		➢ In vitro chemosensitivity testing revealed synergistic chemotherapy combinations between classic chemotherapeutic agents.
		➢ In vitro chemosensitivity testing for classic chemotherapeutic agents matched with clinical response to treatment. ➢ Contrary to non-first line chemotherapy agents (gemcitabine and docetaxel), culture-based NGS could not predict response to TKI treatment.
Song et al. [31]	Cell blocks	➢ EGFR mutations and ALK rearrangements were detected in 68/123 (55.3%) and 11/123 (9%) patients, respectively, while the EGFR wild-type was associated with a PD-L1 IHC score ≥ 50%

NGS: next-generation sequencing, PFS: progression free survival, TKI: tyrosine kinase inhibitor, PARP: poly ADP ribose polymerase, sDNA: sediment DNA.

**Table 5:** The role of NGS in malignant pleural effusions from different types of cancers (other than lung).

First Author/Reference	Cancer	Pleural Fluid Material	Summary of Findings
Yang et al. [39]	Lung, Breast, GI/Pancreas, Primary peritoneal	Supernatants	➢ Pleural fluid supernatant NGS showed concordant genotype with NGS performed on lymph node FNA cell block in a patient with TNBC who underwent chemotherapy in between.
			➢ Pleural fluid supernatant NGS showed concordant genotype with NGS performed on tumor tissue in a patient with CRC who underwent targeted therapy in between.
Shah et al. [53]	Ovarian	Cytospins	➢ Pleural fluid NGS had the least DNA input but highest coverage compared to frozen tumor tissue, FFPE tumor tissue, and matched normal blood from patients with a metastatic ovarian high-grade serous carcinoma.
			➢ Pleural fluid NGS showed the highest TP53 MAFs in two patients.
Xu et al. [54]	Medulloblastoma	N/A	➢ While undetectable in the original tumor tissue, pleural effusion NGS revealed a 17q gain in a patient with medulloblastoma at the time of recurrence.
Zhou et al. [55]	Gastric	Supernatants	➢ Concordant ATM (indel, frameshift, SNV, fusion), MET, and SMAD3 mutations were detected in pleural fluid, plasma, and ascites NGS from an advanced gastric cancer patient.

NSCLC: non-small cell lung cancer, NGS: next-generation sequencing, TNBC: triple negative breast cancer, CRC: colorectal cancer, FFPE: formalin-fixed, paraffin-embedded, FNA: fine-needle aspiration, ATM: ataxia telangiectasia-mutated gene, SNV: single nucleotide variant, MET: mesenchymal to epithelial transition factor, MAF: mutation allele frequency.
